# Effectiveness of poultry litter amendments on bacterial survival and *Eimeria* oocyst sporulation

**DOI:** 10.14202/vetworld.2018.1064-1073

**Published:** 2018-08-06

**Authors:** Essam S. Soliman, Nahla H. Sallam, Eman M. Abouelhassan

**Affiliations:** 1 Department of Animal Hygiene, Zoonosis and Animal Behavior, Faculty of Veterinary Medicine, Suez Canal University, Ismailia, 41522, Egypt; 2 Department of Parasitology, Faculty of Veterinary Medicine, Suez Canal University, Ismailia, 41522, Egypt

**Keywords:** *charcoal*, *Eimeria*, *Escherichia coli*, litter, meta-bisulfide, *Salmonella*, superphosphate

## Abstract

**Aim:**

Broilers’ optimum performance in response to their genetic potential depends on litter environment which is ideal for bacterial survival and coccidian oocyst sporulation. An *in vitro* evaluation was conducted for the effectiveness of superphosphate, meta-bisulfide, and charcoal litter amendments in minimizing *Escherichia coli* O157:H7 and *Salmonella* Typhimurium survival, *Eimeria* oocyst count, and sporulation.

**Materials and Methods:**

Three groups of 16 litter trays were prepared and inoculated with *E. coli* O157:H7, *S*. Typhimurium, and *Eimeria* non-sporulated oocyst. A set of four trays in each group was designed for each one of the chemical amendments. A total of 720 litter samples were collected and examined for bacterial counts, *Eimeria* oocyst count, and sporulation during the experimental period (35 days).

**Results:**

Litter moisture and pH revealed a highly significant (p<0.001) reduction in all treated litter trays compared to control. Total bacterial count (TBC), total *Enterobacteriaceae* count, and *S*. Typhimurium count showed a highly significant (p<0.001) reduction in meta-bisulfide-treated trays compared to other amendments and positive control. Meanwhile, *Eimeria* oocyst count and sporulation revealed a highly significant (p<0.001) reduction in superphosphate, meta-bisulfide, and charcoal-treated trays, respectively. Temperature revealed a highly significant (p<0.001) weak positive correlation with pH of all inoculated trays, a highly significant (p<0.001) weak negative correlation with moisture percentage of *E. coli* O157:H7 and *S*. Typhimurium inoculated trays, and a highly significant (p<0.001) weak negative correlation with TBC. Meanwhile, relative humidity revealed significant (p≤0.005) weak positive correlation with moisture percentage of *E. coli* O157:H7 inoculated trays.

**Conclusion:**

The study concluded that regular usage with periodical reapplication of litter amendments as meta-bisulfide or superphosphate in poultry farms is one of the indispensable managemental and preventive measures for minimizing bacterial survival and inhibiting *Eimeria* oocyst maturation and sporulation.

## Introduction

Litter in poultry farms is a representative ecosystem in which birds remain in contact for almost all of their life. Litter carry important characteristic such as: moisture absorption [1], thermal insulation, protection, and reduction of ammonia [2]. Type of litter material including hay, straw, wood shaving, sawdust, and rice hull can exert an impact on microbial colonization and influence the growth and performance of birds [3].

Litter quality in poultry farms is a major concern, and it may act as a potential vehicle and source for the transmission of pathogenic microorganisms depending on the prevailing temperature, humidity, pH, and moisture content. Litter may allow the survival of many microorganisms incriminated in viral diseases as avian influenza, gumboro, reovirus, laryngotracheitis, and bronchitis; bacterial diseases as colibacillosis and salmonellosis; fungal diseases as ­aspergillosis and mycoses; and parasitic diseases caused by roundworms, tapeworms, and coccidia that depend on wet litter to provide the proper environment for oocysts to sporulate [4].

Moisture and temperature represent the two most important factors affecting fecal coliform survival. Some stains of coliform as *Escherichia* coli O157:H7 can survive in broilers’ litter for 42-49 days at 37°C, for 49-56 days at 22°C, and for 63-70 days at 5°C [5]. Litter in broiler farms can be considered as one of the most favorable media for the growth and transmission of *Salmonella* Typhimurium, increased litter pH, and moisture percentage presented optimal conditions for the increased survival of *S*. Typhimurium. Coccidiosis is one of the most important parasitic diseases in poultry industry [6], and it caused highly economic losses in poultry production represented in mortalities, morbidities, and the cost of preventative or therapeutic drugs and vaccinations [7]. Coccidian mortalities ranked as the second after viral disease mortalities [8]. Control of coccidiosis is usually depended on the use of anticoccidial drugs in feed and litters to prevent and minimize the developmental stages of the *Eimeria* [9].

Chemical litter amendments have numerous benefits as lowering litter pH, prevent transformation of nitrogen into ammonia [10], reduce moisture percentage, absorb odors [11], enhance litter chemical composition, and inhibit enzymatic production and microbial growth. Proper broilers’ farm preparation, chemical litter amendment correct application, and litter management are proper influences which have to be fulfilled to obtain maximum effectiveness.

The present study aimed for evaluating and comparing the effectiveness of some chemical litter amendments (superphosphate, meta-bisulfide, and charcoal) on rates of bacterial survival (*E. coli* O157:H7 and *S*. Typhimurium) and *Eimeria tenella* count and sporulation in poultry litter.

## Materials and Methods

### Ethical approval

The international, national, and institutional applicable guidelines were followed in care and use of birds. The number of birds, as well as the un-necessary repetition, was minimized to avoid excessive stress, discomfort, and suffering. Birds’ requirements were completely satisfied (physiological, psychological, and nutritional).

### Experimental design

Three groups of 16 trays (four sets of four replicates per each inoculum), each 1 m^3^ (3×4×4, 1 m^3^), were prepared. Each tray was filled with poultry litter (Hay). All trays were sterilized by autoclaving at 121°C/20 min. The sterilization was confirmed by sampling 3 g randomly from the litter depth of each tray and added to 27 mL buffered peptone water, mixed thoroughly, and then filtrated; the filtrate tubes were incubated at 37°C for 24 h. About 10 μL from each tube was dropped onto a standard plate count agar (SPCA) and let to dry and incubated at 37°C for 24 h to demonstrate a state of freedom from microbial contamination. Litter trays were supplemented with fresh poultry dropping (500 g/tray) after being sterilized by autoclaving at 121°C/20 min as a source of organic matter to simulate the biological conditions in poultry farms; the supplementation was repeated twice weekly.

### Litter amendments

A set of four litter trays in each group was designed for each one of the chemical amendments. In each group, the first set of four trays was treated with superphosphate, the second set of four trays was treated with meta-bisulfide, the third set of four trays was treated with charcoal, and the fourth set of four litter trays was used as control positive (inoculated untreated trays). Superphosphate, meta-bisulfide, and charcoal were added by 0.5 g, 0.05 g, and 0.05 g/m^2^, respectively. The amendments were added once at the beginning of the experiment, which was designed to last for 35 days.

### Bacterial cultures and coccidium oocyst

*E. coli* O157:H7 2.5×10^7^ colony-forming units (CFU)/mL culture was purchased from Animal Health Research Institute, Dokki, and propagated using MacConkey broth at 44°C/24 h. 10 μL was transferred aseptically and dropped onto eosin methylene blue (EMB) agar and incubated at 37°C/24 h [12]. Metallic green colonies were counted and picked up. The sixteen trays of the 1^st^ group were inoculated with *E. coli* O157:H7 1.6×10^8^ CFU/mL and mixed thoroughly to ensure even distribution.

*S*. Typhimurium lyophilized vial 2.2×10^4^ CFU was purchased from Animal Health Research Institute, Dokki, reconstituted and propagated using tetrathionate broth, and incubated at 37°C/24 h, and the pre-enrichment was repeated daily for 7 successive days. About 10 μL was dropped under complete aseptic conditions onto CHROMagar [12], and the plates were incubated at 37°C/24 h. Pink colonies were counted and pick up. The sixteen trays of the second group were inoculated with *S*. Typhimurium 5.8×10^7^ CFU/mL and mixed thoroughly.

*E. tenella* sporulated oocyst 1×10^4^ oocyst per gram (OPG) in potassium dichromate 2.5% suspension was provided kindly by Animal Health Research Institute, Ismailia. *E. tenella* sporulated oocyst suspension was washed 3 times with centrifugation, resuspended in distilled water, and propagated experimentally in broilers. Ten broilers of two-weeks old were purchased from Ismailia-Egypt Poultry Co, housed in metal galvanized battery, given *ad libitum* access to water, and supplied with a standard corn-soybean basal diet to meet their dietary requirements according to the National Research Council, [13]. Birds were challenged with *E. tenella* sporulated oocyst 1×10^4^ OPG by oral gavage and kept under observation for the development of clinical manifestation (bloody diarrhea). The fecal material was collected daily for 7 successive days from the onset of clinical manifestation and examined. Positive fecal material revealed the presence of non-sporulated *E. tenella* oocysts was kept in sterilized screw capped bottle in the refrigerator at 4°C. Non-sporulated *E. tenella* oocyst 3×10^4^ OPG was added to the sixteen trays of the third group.

### Sampling and measurements

A total of 720 Litter samples were collected during the study period (three samples weekly from each tray for 5 consecutive weeks). Litter samples were collected by receiving a handful amount from the depth of the litter tray in polyethylene bags. Samples were preserved at 4°C for bacteriological and parasitological examination.

### Microclimatic measures

Microclimatic temperature and relative humidity were recorded daily during the entire length of the experiment using Clock and Hygro-Thermometer (Boeco Germany, BOE 325).

### Litter chemical examination

Litter pH was recorded daily using Jenway 370 pH meter. Meanwhile, moisture content was determined 3 times weekly by weighting litter samples (W1) using JJ223BC 0.001 g 220 g electronic analytical balance and dried in LABTECH digital hot air oven at 100°C for 36-48 h, and the dry weight (W2, two equal successive weights) was subtracted from the initial weight (W1) to detect changes in litter’s moisture.

### Bacteriological examination

Litter samples were prepared by adding 3 g sample to 27 mL buffered peptone water, and the mixture was vortexed and filtered. 1 mL filtrate was transferred aseptically to a sterilized tube containing 9 mL of physiological saline. Ten-fold serial dilutions up to 10^−6^ were prepared. Total bacterial count (TBC), total *Enterobacteriaceae* count (TEC), and *S*. Typhimurium count were applied using a drop plate technique [12,14]. SPCA was inoculated with different filtrate concentrations for TBC at 37°C for 24-48 h, EMB agar was used for TEC at 37°C for 24-48 h, and CHROMagr for *S*. Typhimurium count at 37°C for 24-48 h. Plates showed 30-300 CFU were counted using Dark-field Colony Counter [15].

### Parasitological examination

Samples were examined by sugar flotation technique [16,17] for the detection of oocyst. The quantification of oocyst was carried out using McMaster[18], multiplied by the dilution factor (100×), and expressed by OPG.

### Statistical analysis

Statistical analysis was carried out using the Statistical Package for the Social Sciences [19] and statistical analysis system [20]. *E. coli* O157:H7, *S*. Typhimurium, and *Eimeria* oocyst counts were expressed as logarithm using Microsoft Excel. The obtained data were analyzed statistically using multifactorial analysis of variance with multivariate general linear model procedures (generalized linear modeling) for treated and inoculated untreated trays (positive control), time, temperature, relative humidity, and their interactions [21].

## Results

Meta-bisulfide and superphosphate litter amendments revealed as shown in Table-1 a highly significant (p<0.001) reduction in litter moisture ­percentage rather than charcoal compared to positive control (untreated) trays. Meta-bisulfide revealed a highly significant (p<0.001) reduction of moisture percentage (Table-1) at the 3^rd^ week in all inoculated litter trays, superphosphate showed a highly significant (p<0.001) reduction of moisture percentage (Table-1) at the 3^rd^ week in *E. coli* O157:H7 and *S*. Typhimurium inoculated trays and at the 4^th^ week in *E. tenella* inoculated trays, and charcoal revealed a highly significant (p<0.001) reduction of moisture percentage (Table-1) at the 4^th^ week in all inoculated litter trays.

**Table-1:** Moisture percentage (mean±SE) of treated and untreated litter inoculated with *E. coli* O157:H7, *S.* Typhimurium*,* and *E. tenella*.

Litter amendments	Time/week	Litter inoculums		

		*E. coli* inoculum	*Salmonella* inoculum	*E. tenella*inoculum
Superphosphate		31.80^c^±0.16	30.34^d^±0.05	30.67^c^±0.18
Meta-bisulfide		31.59^c^±0.33	31.14^c^±0.21	30.92^c^±0.19
Charcoal		35.31^b^±0.18	36.05^b^±0.22	34.21^b^±0.15
Control positive		41.68^a^±0.26	41.34^a^±0.25	39.88^a^±0.21
p-value		0.001	0.001	0.001
Amendment* time				
Superphosphate	1^st^	29.83^c^±1.92	30.12^b^±1.72	29.30^c^±1.49
	2^nd^	33.16^b^±1.71	31.95^a^±0.80	32.62^a^±1.13
	3^rd^	34.29^a^±1.19	31.91^a^±0.82	31.33^b^±1.04
	4^th^	29.91^c^±1.52	27.37^c^±1.24	29.37^c^±2.18
	5^th^	29.18^c^±1.48	28.00^c^±1.15	29.19^c^±2.01
Metabisulfide	1^st^	31.62^b^±0.76	31.66^c^±0.76	31.12^b^±0.85
	2^nd^	34.95^a^±3.51	33.37^a^±5.20	34.04^a^±3.58
	3^rd^	31.37^b^±0.82	32.54^b^±3.98	31.33^b^±0.48
	4^th^	28.41^c^±0.58	27.00^d^±1.25	27.20^c^±1.58
	5^th^	28.40^c^±0.49	27.14^d^±1.05	27.28^c^±1.18
Charcoal	1^st^	37.12^a^±0.85	39.66^a^±2.07	33.16^c^±1.71
	2^nd^	35.37^c^±0.68	34.79^c^±3.20	34.25^b^±0.94
	3^rd^	36.87^b^±0.85	36.91^b^±1.61	36.91^a^±0.77
	4^th^	31.87^d^±0.85	32.83^d^±1.12	32.54^d^±1.17
	5^th^	31.18^d^±0.81	32.71^d^±1.01	32.44^d^±1.05
Control positive	1^st^	45.50^a^±3.03	44.41^b^±1.38	38.45^b^±0.50
	2^nd^	44.75^b^±1.22	45.50^a^±2.32	40.66^a^±0.70
	3^rd^	38.45^c^±2.97	37.50^c^±0.51	40.00^a^±1.53
	4^th^	38.04^c^±0.85	37.95^c^±0.85	40.41^a^±1.50
	5^th^	38.16^c^±0.79	37.66^c^±0.81	40.44^a^±1.54
p-value		0.001	0.001	0.001

Means carrying different superscripts in the same column are significantly different at (p≤0.05) or highly significantly different at (p<0.01). Means carrying the same superscripts in the same column are non-significantly different at (p<0.05). SE: Standard error, *E. coli*=*Escherichia coli, S.* Typhimurium=*Salmonella* Typhimurium*, E. tenella=Eimeriatenella*

Litter pH as shown in Table-2 revealed a highly significant (p<0.001) overall decrease in meta-bisulfide, superphosphate, and charcoal-treated litter trays, respectively. Superphosphate, meta-bisulfide, and charcoal as shown in Table-2 produced a highly significant (p<0.001) decrease of pH from the 1^st^ week, and as the experiment proceeds, pH showed a constant increase without achieving the neutral pH once again.

**Table-2:** pH (mean±SE) of treated and untreated litter inoculated with *E. coli* O157:H7, *S.* Typhimurium*,* and *E. tenella*.

Litter amendments	Time/week	Litter inoculums		

		*E. coli* inoculum	*Salmonella* inoculum	*E. tenella* inoculum
Superphosphate		5.73^b^±0.44	5.67^c^±0.04	5.72^b^±0.05
Meta-bisulfide		5.35^c^±0.24	5.31^d^±0.03	5.33^c^±0.12
Charcoal		5.80^b^±0.63	5.95^b^±0.04	5.73^b^±0.09
Control Positive		7.73^a^±0.81	6.51^a^±0.05	6.32^a^±0.04
P-value		0.001	0.001	0.001
Amendment* time				
Superphosphate	1^st^	4.53^d^±0.70	4.67^d^±0.46	4.67^c^±0.44
	2^nd^	5.56^c^±0.28	5.84^c^±0.26	5.69^b^±0.43
	3^rd^	6.35^b^±0.45	6.05^b^±0.56	6.30^a^±0.50
	4^th^	6.49^a^±0.42	6.11^a^±0.31	6.24^a^±0.21
	5^th^	6.41^a^±0.41	6.10^a^±0.28	6.23^a^±0.22
Meta-bisulfide	1^st^	4.65^d^±0.40	4.46^d^±0.61	4.44^d^±0.44
	2^nd^	5.21^c^±0.24	5.31^c^±0.28	5.46^c^±0.28
	3^rd^	5.64^b^±0.21	5.49^b^±0.18	5.58^b^±0.45
	4^th^	5.89^a^±0.29	5.99^a^±0.28	5.85^a^±0.30
	5^th^	5.86^a^±0.23	5.98^a^±0.23	5.82^a^±0.28
Charcoal	1^st^	5.51^d^±0.08	5.16^c^±0.61	5.46^c^±0.94
	2^nd^	5.66^c^±0.20	5.94^b^±0.50	5.62^b^±0.43
	3^rd^	5.78^b^±0.17	6.39^a^±0.64	5.70^b^±0.22
	4^th^	6.27^a^±0.44	6.31^a^±0.26	6.16^a^±0.32
	5^th^	6.22^a^±0.32	6.30^a^±0.18	6.12^a^±0.16
Control positive	1^st^	6.92^d^±0.56	6.67^b^±0.45	5.73^d^±0.70
	2^nd^	7.77^c^±0.80	6.86^a^±0.63	6.17^c^±0.77
	3^rd^	8.26^a^±0.10	6.29^c^±0.17	7.07^a^±0.57
	4^th^	7.95^b^±0.31	6.23^c^±0.29	6.31^b^±0.14
	5^th^	7.92^b^±0.32	6.22^c^±0.35	6.30^b^±0.17
p-value		0.001	0.001	0.001

Means carrying different superscripts in the same column are significantly different at (p≤0.05) or highly significantly different at (p<0.01). Means carrying the same superscripts in the same column are non-significantly different at (p<0.05). SE: Standard error, *E. coli*=*Escherichia coli, S.* Typhimurium=*Salmonella* Typhimurium*, E. tenella=Eimeriatenella*

A highly significant (p<0.01) reduction of total bacterial and *S*. Typhimurium counts was detected (Table-3) in litter trays treated with meta-bisulfide, superphosphate, and charcoal, respectively, compared to control positive trays. Meanwhile, TEC revealed a highly significant (p<0.01) reduction in meta-bisulfide-treated litter trays, with no significant differences between superphosphate and charcoal-treated litter trays.

**Table-3:** Logarithmic bacterial counts (mean±SECFU/mL) in treated and untreated poultry litter inoculated with *E. coli* O157:H7 and *S.* Typhimurium.

Litter Amendments	Time/week	Log TBC CFU/ml	Log TEC CFU/ml	Log *Salmonella* count CFU/ml
Superphosphate		6.079^c^±0.052	3.500^b^±0.139	2.637^c^±0.077
Meta-bisulfide		4.935^d^±0.110	2.450^c^±0.088	2.179^d^±0.081
Charcoal		6.279^b^±0.098	3.786^b^±0.112	3.916^b^±0.093
Control positive		6.804^a^±0.116	5.290^a^±0.359	5.133^a^±0.132
p-value		0.001	0.001	0.001
Amendment* time				
Superphosphate	1^st^	6.940^a^±0.104	6.440^a^±0.081	4.469^a^±0.124
	2^nd^	6.619^b^±0.028	3.617^b^±0.102	2.791^b^±0.012
	3^rd^	5.501^c^±0.013	3.064^c^±0.221	2.549^c^±0.024
	4^th^	5.255^d^±0.098	0.880^d^±0.312	0.738^d^±0.343
	5^th^	3.306^e^±0.018	0.000^e^±0.312	0.000^e^±0.343
Meta-bisulfide	1^st^	5.692^a^±0.016	2.394^c^±0.102	2.097^c^±0.041
	2^nd^	5.517^b^±0.156	3.900^a^±0.312	3.336^a^±0.414
	3^rd^	4.823^c^±0.330	3.506^b^±0.432	3.284^b^±0.310
	4^th^	3.706^d^±0.083	0.000^d^±0.000	0.000^d^±0.000
	5^th^	1.517^e^±0.051	0.000^d^±0.000	0.000^d^±0.000
Charcoal	1^st^	5.843^d^±0.122	4.908^a^±0.251	4.860^a^±0.320
	2^nd^	6.133^c^±0.589	4.800^b^±0.223	3.879^b^±0.031
	3^rd^	6.733^a^±0.079	3.510^c^±0.129	3.710^c^±0.231
	4^th^	6.408^b^±0.178	1.925^d^±0.038	3.213^d^±0.031
	5^th^	5.751^d^±0.118	1.780^d^±0.321	2.117^e^±0.105
Control positive	1^st^	7.609^a^±0.284	6.586^a^±0.341	6.155^a^±0.052
	2^nd^	6.336^d^±0.089	5.382^b^±0.112	6.186^a^±0.084
	3^rd^	6.730^b^±0.158	4.657^c^±0.241	4.060^b^±0.139
	4^th^	6.542^c^±0.030	4.533^c^±0.131	4.128^b^±0.039
	5^th^	6.735^b^±0.036	6.465^c^±0.124	4.133^b^±0.124
p-value		0.001	0.001	0.001

Means carrying different superscripts in the same column are significantly different at (p≤0.05) or highly significantly different at (p<0.01). Means carrying the same superscripts in the same column are non-significantly different at (p<0.05). SE: Standard error, TBC=Total bacterial count, TEC=Total Enterobacteriaceae count, CFU=Colony-forming unit, *E. coli=Escherichia coli*, *S. Typhimurium=Salmonella* Typhimurium

On a weekly basis, superphosphate revealed (Table-3) a uniform pattern of highly significant (p<0.001) reduction in total bacterial, *Enterobacteriaceae*, and *S*. Typhimurium counts from the 1^st^ week. Meta-bisulfide as shown in Table-3 revealed a highly significant (p<0.001) reduction in TBC from the 1^st^ week and in total *Enterobacteriaceae* and *S*. Typhimurium counts from the 2^nd^ week and achieved zero counts at the 4^th^ week compared to positive control. Charcoal revealed, in Table-3, a uniform pattern of a highly significant (p<0.001) reduction in *Enterobacteriaceae* and *S*. Typhimurium counts from the 1^st^ week, while TBC did not respond for the amendment until the 4^th^ week compared to the positive control trays.

**Figure-1:**
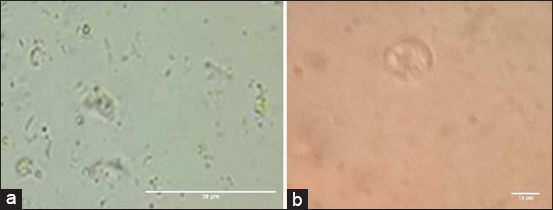
Light microscopic picture of *Eimeria tenella* oocyst in superphosphate treated litter. (a) Remnant parts of *E. tenella* oocyst. (b) Broken *E. tenella* oocyst.

**Figure-2:**
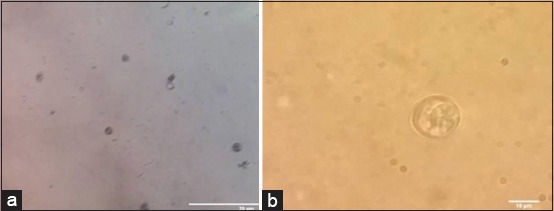
Light microscopic picture of *Eimeria tenella* oocyst in positive control (inoculated untreated litter). (a) Non-sporulated *E. tenella* oocyst. (b) Sporulated *E. tenella* oocyst.

**Figure-3:**
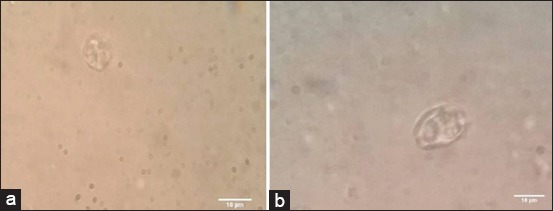
Light microscopic picture of *Eimeria tenella* oocyst in meta-bisulfide-treated litter. (a) Disrupted shaped *E. tenella* oocyst. (b) Broken *E. tenella* oocyst.

**Figure-4:**
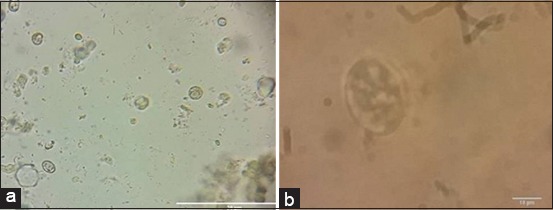
Light microscopic picture of *Eimeria tenella* oocyst in charcoal-treated litter. (a) Intact non-sporulated *E. tenella* oocyst. (b) Sporulated *E. tenella* oocyst.

*E. tenella* oocyst sporulation and count as shown in Table-4 and Figure-1 revealed a highly significant (p<0.001) extinction and destruction of *Eimeria* oocyst from the 1^st^ week in superphosphate-treated trays compared to intact non-sporulated *Eimeria* oocyst in control positive untreated trays (Figure-2). Meta-bisulfide revealed, in Table-4 and Figure-3, a highly significant (p<0.001) reduction in *E. tenella* oocyst counts with cessation of sporulation from the 1^st^ week, and zero counts with complete destruction of all *Eimeria* oocyst were achieved by the 4^th^ week compared to control positive (inoculated untreated litter) in Figure-2. Meanwhile, charcoal revealed a highly significant (p<0.001) reduction in *E. tenella* oocyst count (Table-4 and Figure-4) from the 1^st^ week and achieved zero counts at the 4^th^ week, but it did not have the ability to cease the oocyst sporulation activity in litter that continued until the end of the 3^rd^ week compared to control positive (inoculated untreated litter) in Figure-2 which revealed the intact non-sporulated *E. tenella* oocyst.

**Table-4:** *Eimeria* oocyst sporulation and logarithmic count (mean±SE OPG) in treated and untreated poultry litter inoculated with *E. tenella*.

Time/weeks	Parameter	Litter amendments			

		Superphosphate	Meta-bisulfide	Charcoal	Positive control
1^st^	Oocyst	Negative	NS	NS	NS
	Count/OPG	0^a^	3.669^a^	3.845^a^	3.903^a^
2^nd^	Oocyst	Negative	NS	S	S
	Count/OPG	0^a^	3.301^b^	3.000^b^	3.301^b^
3^rd^	Oocyst	Negative	NS	S	S
	Count/OPG	0^a^	3.000^c^	3.000^b^	3.000^c^
4^th^	Oocyst	Negative	Negative	Negative	Negative
	Count/OPG	0^a^	0^d^	0^c^	0^d^
5^th^	Oocyst	Negative	Negative	Negative	Negative
	Count/OPG	0^a^	0^d^	0^c^	0^d^

Means carrying different superscripts in the same column are significantly different at (P≤0.05) or highly significantly different at (p<0.01). Means carrying the same superscripts in the same column are non-significantly different at (p<0.05). NS=Non-sporulated oocyst, S=Sporulated oocyst, OPG=Oocyst per gram, SE: Standard error, *E. tenella=Eimeriatenella*

Temperature revealed highly significant weak positive correlations (r=0.186, 0.176, 0.281, p<0.001) with pH of *E. coli* O157:H7*, S*. Typhimurium, and *E. tenella* inoculated litter trays, respectively, as shown in Table-5. Although temperature revealed highly significant weak negative correlations (r=−0.207, −0.210, p<0.001) with moisture percentage of *E. coli* O157:H7 and *S*. Typhimurium inoculated litter trays (Table-5) and a highly significant weak negative correlation (r=−0.312, p<0.001) with TBC (Table-6). Relative humidity in Table-5 revealed a significant weak positive correlation (r=0.103, p<0.001) with moisture percentage of *E. coli* O157:H7 inoculated litter trays.

**Table-5:** Correlation coefficient between chemical characteristics of litter inoculated with *E. coli* O157:H7, *S.* Typhimurium*,* and *E. tenella* with ambient temperature (above diagonal) and relative humidity (below diagonal).

r	Temperature	*E. coli* inoculum		*Salmonella* inoculum		*Eimeria* inoculum	
		
		pH	Moisture	pH	Moisture	pH	Moisture
RH	1	0.186**	−0.207**	0.176**	−0.210**	0.261**	0.063
*E. coli* inoculum							
pH	−0.059	1	0.564**	0.683**	0.469**	0.680**	0.659**
Moisture	0.103*	0.564**	1	0.474**	0.910**	0.217**	0.821**
*Salmonella* inoculum							
pH	−0.080	0.683**	0.474**	1	0.384**	0.597**	0.475**
Moisture	0.076	0.469**	0.910**	0.384**	1	0.129*	0.807**
*Eimeria* inoculum							
pH	0.034	0.680**	0.217**	0.597**	0.129**	1	0.332**
Moisture	0.042	0.659**	0.821**	0.475**	0.807**	0.332**	1

r = Pearson’s correlation,

** Correlation is significant at the 0.01 level.

* Correlation is significant at the 0.05 level.

^NS^Correlation is non−significant at the 0.05 level. *E. coli=Escherichia coli, E. tenella=Eimeriatenella, S.* Typhimurium=*Salmonella* Typhimurium, SE: Standard error

**Table-6:** Correlation coefficient between bacterial and coccidian oocyst load in litter inoculated with *E. coli* O157:H7, *S.* Typhimurium, and *E. tenella* with ambient temperature (above diagonal) and relative humidity (below diagonal).

r	Temperature	TBC	TEC	*Salmonella* count	*Eimeria* oocyst
RH	1	−0.312**	−0.043	−0.078	0.095
TBC	0.122	1	0.699**	0.704**	0.235**
TEC	0.117	0.699**	1	0.903**	0.367**
*Salmonella* count	0.090	0.704*	0.903**	1	0.451**
*Eimeria* oocyst	0.117	0.235**	0.367**	0.451**	1

r=Pearson’s correlation,

** Correlation is significant at the 0.01 level.

* Correlation is significant at the 0.05 level.

^NS^Correlation is non-significant at the 0.05 level. TBC=Total bacterial count, TEC=Total *Enterobacteriaceae* count, *S.* Typhimurium=*Salmonella* Typhimurium

## Discussion

Deep litter system is one of the most common housing systems in broilers’ production worldwide. Litter is a mixture of pathogen-free bedding material (hay, straw, wood shaving, sawdust, and rice hull), bird’s excreta that contained high levels of nitrogen from dietary protein, feathers, and wasted feed. Thus, by the end of each broilers’ cycle, litter becomes seeded with high microbial load up to 10^10^ CFU/g [22]. A variety of pathogenic microorganisms that affect bird’s growth and performance can be isolated from broilers’ litter as *Actinobacillus* spp.*, Campylobacter* spp., *Clostridium* spp.*, Corynebacterium* spp., *Escherichia coli, Listeria* spp., *Mycobacterium* spp.*, Salmonella* spp., *Staphylococcus* spp., and *Streptococcus* spp [23]. In an epidemiological survey in broilers’ farms, Soliman *et al*. [24] isolated and identified a wide variety of bacterial microorganisms from broilers’ litter including *E. coli, Salmonella* spp., *Klebsiella oxytoca, Pseudomonas aeruginosa, Shigella* spp., *Citrobacter* spp.*, Proteus vulgaris, Streptococcus faecalis, Streptococcus pneumoniae*, and *Staphylococcus aureus*. Microbial load (aerobic, anaerobic, and coliform) usually increased in the superficial layers of poultry litter by the direct deposition of fecal material that contains a high level of bacterial and protozoal organisms and decreased as litter depth increased [25]. Chen and Jiang [26] stated that microbial concentration in litter can achieve 10^10^ CFU/g, from which Gram-positive bacteria represent about 90%. Microorganisms as *Clostridium, Escherichia coli, Salmonella, Streptococcus, Staphylococcus*, and *Listeria* can exert different metabolic activities in litter causing interference with growth and production of poultry.

Litter conditions are the principal key to overall management of poultry farm. Broilers’ litter is naturally a hostile environment for survival and persistence of many microorganisms as it becomes dry and heats up rapidly in normal and securely managed broilers’ houses. Litter pH, moisture percentage, temperature, and relative humidity levels are responsible for microbial growth in poultry litter [27]. Daí Pra *et al*. [28] found that an increase in the litter’s temperature might contribute a significant reduction in microbial load. Heat treatment of litter studied by Stringfellow *et al*. [29] caused an increase in litter pH that contributed a significant reduction in microbial colonization of *S*. Typhimurium. Wilkinson *et al*. [30] found a great reduction in *E. coli* and *S*. Typhimurium counts by 99% in 1 h at 55 and 65°C, and they were able to survive for longer periods at lower temperature (35°C) accompanied with high moisture percentage (65%) in a laboratory trial. Meanwhile, in field trials, they examined four broilers’ litter samples and found that initial fecal coliform counts were 5.07, 5.21, 4.64, and 5.91 log_10_ CFU/g, these counts declined by 96% after 2-16 weeks to 3.65, 3.78, 3.20, and 4.47 log_10_ CFU/g, respectively.

Chemical and biological litter amendments enforced itself into poultry industry in many shapes as acidifying agents that reduce litter pH, clay-based products that absorb odors, especially ammonia, and microbial inhibitors that inhibit enzyme synthesis, microbial growth, and multiplication. Variables such as litter stocking, litter moisture, litter pH, breed, temperature, relative humidity, and concurrent disease threats can influence the choice of litter amendment type. The choice of chemical litter amendments was more successful compared to other forms of amendments, as it reduces litter pH and moisture percentage creating unfavorable conditions for bacterial and viral survival as well as coccidian sporulation and maturation [31]. Chemical amendments cannot provide solutions for inadequate ventilation, small air inlet, and high bird stocking density. That is why broilers’ houses should be provided with strict management system before application of these amendments.

Our results indicated that meta-bisulfide and superphosphate were relatively acidic amendments and, when applied to litter trays, contributed a sharp drop in litter pH up to 5.6:5.7 in superphosphate-treated trays and 5.3 in meta-bisulfide-treated trays. Meanwhile, Charcoal was able to decrease litter pH up to 5.7:5.9 compared to superphosphate and meta-bisulfide. Chemical amendments were able to maintain such acidic pH for the entire duration of the experiment with a little fluctuation as a trial to achieve a neutral pH starting from the 3^rd^ week up to the end of the experiment. These results agreed with those reported by McWard and Taylor [32], who revealed that poultry guard acidifying clay, alum, and sodium bisulfate (SBTL) significantly reduced litter pH and ammonia volatilization from the litter. The results agreed with those of Medeiros *et al*. [33] studied the influence of superphosphate in litter amendment using 0, 5, 10, 15, 20, and 25% in litters from four cycles and found that pH sharply reduced from 8.4 to 5.8.

Chemical amendment application in litter trays caused a dramatic decline in moisture percentage, which was more prominent in superphosphate and meta-bisulfide rather than charcoal-treated trays. Our results agreed with those of Sahoo *et al*. [34], who examined the efficiency of alum sulfate (ATL at dose rate of 90 g.f^-2^) and sodium bisulfate (SBTL at dose of 25 g.f^-2^) litter amendments on litter quality, broilers’ performance, carcass characteristics, and broilers’ welfare during winter season; they found that litter treated with SBTL had more moisture and lower pH than that treated with ATL compared to control; the acidification of litter contributed to a great improvement in litter quality and enhanced productivity and broilers’ welfare. Furthermore, the results agreed with those of Schneider *et al*. [35], who found that the addition of zeolite 10% to sawdust litter was able to reduce litter moisture contents up to 32% so that it can be used for three consecutive broilers’ flocks safely. The current results also agreed with those of Soliman and Hassan [36], who reported a highly significant (p<0.001) reduction of aerial ammonia concentration in both laboratory and field trials caused by a significant reduction in litter moisture percentage and pH in superphosphate and sodium meta-bisulfide-treated litter, respectively.

*E. coli* have a great ability to survive in different environments including air, water, manure, and soil with great possibilities to migrate between these habitats [37]. The survival capabilities of *E. coli* in litter depend on numerous factors including energy and nutrients availability, pH, moisture, and temperature. Van Elsas *et al*. [38] stated that *E. coli* O157:H7 have great survival capabilities for long periods (130 days) in manure soil at dry conditions (1% moisture) and 18°C. *S*. Typhimurium can survive for significantly more extended periods compared to *E. coli*, and this might be attributed to the genome structure represented in 1-4% increase in guanine plus cytosine content in *S*. Typhimurium [38].

Total bacterial, *Enterobacteriaceae* and *S*. Typhimurium counts in our study were significantly reduced due to the drastic decline in litter pH and moisture content ensured by the highly significant (p<0.001) correlations between pH and moisture percentage in all inoculated litter trays, making the litter an unfavorable media for microbial survival and growth. The results agreed with those of Soliman *et al*. [39], who found a significant reduction in survival of *S*. Typhimurium caused by a collaboration of some condition including a decline of litter pH, an increase of ambient temperature, and a decrease of relative humidity irrespective to the presence or absence of organic matter source. Bennett *et al*. [40] findings were agreed to our results, as they found that 5%, 10%, and 20% hydrated lime significantly reduced the survival of *Salmonella enteritidis* in *in vitro* experiment after 24 h from the application in poultry litter. On the other hand, Bennett *et al*. [41] did not agree with our results as they revealed that 5% hydrated lime was not able to suppress the survival of *Salmonella* and *Campylobacter*, but it did cause a low significant reduction in the total bacterial aerobic count.

Line and Bailey [42] also agreed with our results and found that aluminum sulfate and SBTL caused a little degree of delay in *Campylobacter* colonization in broiler chicks, while *Salmonella* levels remained unaffected. Vicente *et al*. [43] also agreed with our results and revealed that using low dose (360 g.m^−2^) as well as high dose (720 g.m^-2^) of acidified clay minimized *Salmonella enteritidis* recovery in poultry litter to 0 and 3%, respectively, after 11 days and 23 and 18%, respectively, after 21 days. Lopes *et al*. [44] agreed with our results and found that adding 300 g quicklime per square meter litter caused a significant reduction in CFU counted on brain heart infusion, Chapman, and MacConkey agar by 57.2, 66.9, and 92.1%, respectively, and they contributed this reduction to the decline in litter pH. Furthermore, Soliman and Hassan [36] results agreed with our results as they recorded a highly significant (p<0.001) reduction in total bacterial and TECs with an increase in body weight, performance index, and bursa’s weight in birds raised on superphosphate-treated litter compared to a highly significant (p<0.001) increase in weight gain, spleen, and thymus weight in birds raised on meta-bisulfide-treated litter.

Coccidiosis is one of the most critical threats that face the global poultry industry. Chickens are susceptible to nine *Eimeria* species that belong to phylum *Apicomplexa* [45]. Understanding coccidiosis epidemiology and developing control and preventive measures rely on gaining knowledge about the distribution and structure of *E. tenella* [46].

Our results indicated that the used litter amendments produced high ability to destroy *E. tenella* oocyst directly from the 1^st^ week in superphosphate-treated litter, to reduce *E. tenella* oocyst count with cessation of sporulation and achieving zero count by the 4^th^ week in meta-bisulfide-treated litter, or to reduce *E. tenella* oocyst count without cessation of sporulation and achieving zero count by the 4^th^ week in charcoal-treated litter. The used chemical amendments (superphosphate, meta-bisulfide, and charcoal) were able to modify litter abiotic conditions (pH and moisture content) creating harsh conditions for *Eimeria* oocyst to complete its development and sporulation in poultry litter. Results agreed with those of Fetterer *et al*. [47] who revealed that using 300 µg/ml aqueous concentrations of metam sodium (sodium N-methyldithiocarbamate) for 24 h was able to prevent the sporulation and significantly reduce the viability of *E. tenella*, *Eimeria acervulina*, and *Eimeria maxima* oocyst in poultry litter. Sahoo *et al*. [48] found that treating litter with SBTL was able to reduce *Eimeria* oocyst count to a little extent compared to ATL and control, although, of the numerical values, differences between the two amendments were statistically non-significant.

The results also agreed with Samaha *et al*. [49], who revealed a higher *in vitro* efficiency for ammonium hydroxide 5 and 10% and phenol 10% (99% reduction in count) compared to Eco-Bio (quaternary ammonium and glutaraldehyde combination) and ZixVirox (peracetic acid and hydrogen peroxide combination) against *E. tenella* oocyst under the influence of abiotic conditions as temperature, pH, and presence of organic matter. Meanwhile, Mesa *et al*. [50] observed no reduction in *E. maxima* count in poultry litter when covered with plastic canvas for 8 days.

## Conclusion

Litter amendments as meta-bisulfide, superphosphate, and to little extent charcoal were able to mutate and modify neutral pH and high moisture percentage of litter, which were considered optimum conditions for bacterial survival, protozoal maturation, and sporulation. Application of litter amendments as meta-bisulfide or superphosphate in poultry farms with regular reapplication is indispensable managemental and preventive measure for maintaining the mutated abiotic conditions which reduce bacterial survival and inhibit protozoal maturation, thus maintaining a healthy flock.

## Authors’ Contributions

ESS designed the experimental design and participated in the preparation, execution of the experiment, and writing the manuscript. NHS assisted in laboratory work and participated in the parasitological examination and in writing the manuscript. EMA participated in laboratory work, in the parasitological examination, and in writing the manuscript. All authors read and approved the final manuscript.
